# Research Note: PSE condition is associated with increased apoptotic potential in broiler *pectoralis major* muscle

**DOI:** 10.1016/j.psj.2022.102107

**Published:** 2022-08-04

**Authors:** Boin Lee, Young Min Choi

**Affiliations:** Department of Animal Sciences and Biotechnology, Kyungpook National University, Sangju-Si 37224, South Korea

**Keywords:** cytochrome c, caspases, apoptotic potential, meat quality, chicken breast

## Abstract

This study compared the meat quality and sensory quality characteristics of broiler *pectoralis major* (**PM**) muscle between meat quality classes, including reddish, firm, and non-exudative (**RFN**) and pale, soft, and exudative (**PSE**) conditions. Additionally, we also investigated the associations between the meat quality classes and expression levels of cytochrome c, initiator (caspase 9), and effector caspases (caspase 3 and 7) at the early postmortem period. A total of 135 PM muscles from broilers were used, and meat quality classes were determined according to the pH_24 h_ and lightness values and classified into the RFN (N = 81) and PSE (N = 54) conditions. The PSE breasts showed lower muscle pH_15 min_ and pH_24 h_ values compared to the RFN breasts (*P* < 0.05). A lower lightness value was observed in the RFN group compared to the PSE group (*P* < 0.001), whereas there were no significant differences in redness and yellowness between the groups (*P* > 0.05). The PSE group exhibited higher extent of water loss, including drip loss and cooking loss, compared to the RFN group (*P* < 0.05). For these reasons, samples from the RFN group required less force to breakdown the cooked meat with more moisture in the mouth after chewing compared to samples from the PSE group (*P* < 0.001); however, flavor intensity did not differ between the groups (*P* > 0.05). At 15 min postmortem, all apoptosis-related molecules, including cytochrome c, caspase 9, caspase 3, and caspase 7, were present at higher levels in the PSE group than in the RFN group (*P* < 0.05). These results indicated that higher apoptotic potentials were associated with the development of PSE chicken breasts. Therefore, the variation of meat quality in chicken breast can be explained as being affected by the expression levels of apoptosis-related factors at the early postmortem period.

## INTRODUCTION

One of the important issues in the poultry industry is the occurrence of pale, soft, and exudative (**PSE**) condition meat, which arises as a result of intensive selection for increased muscling, particularly the *pectoralis major* (**PM**) muscle (Owens et al., 2000). This condition occurs in more than 20% of the total production in commercial chicken slaughtering plants in many countries (Grashorn, 2010; Zhu et al., 2012; Karunanayaka et al., 2016). The poultry industry has been working to reduce the incidence of the PSE condition, since it can negatively affect consumer acceptability, meat quality traits, and further processing properties (Kuttappan et al., 2012; Gonzalez, 2020). In general, the development of PSE condition is dependent on the extent of physicochemical changes during the conversion of muscle to meat after bleeding, and the mechanisms responsible for this development are interdependent and extremely complex (Kemp and Parr, 2012). The first phase in this conversion during the postmortem period is the onset of programmed cell death, which is a central homeostatic process. Thus, the biochemical and structural changes caused by the metabolic switch suffered by muscle fibers during apoptosis can influence the meat quality (Lomiwes et al., 2014).

Apoptosis is effectively established by caspases, cysteine aspartate-specific proteases that induce myofibril protein cleavage (Kemp and Parr, 2012). In caspase-mediated apoptosis, it is subdivided into 3 main pathways of activation, including intrinsic, extrinsic, and endoplasmic reticulum-mediated pathways (Kemp and Parr, 2012). The pathway induced by hypoxic/ischemic conditions during postmortem is the intrinsic apoptosis pathway, a form of mitochondrion-centered cell death mediated by the permeabilization of mitochondrial outer membrane (Concannon et al., 2003). Disruption of the mitochondrial membrane leads to the release of cytochrome c, which binds apoptosis protease activating factor 1 in the presence of dATP in the cytoplasm (Concannon et al., 2003). This protein complex, known as the apoptosome, activates caspase 9, triggering a cascade of effector caspases, including caspase 3 and 7 (Concannon et al., 2003). During the postmortem period, these caspase systems are responsible for muscle fiber destruction and affect the meat quality traits of various animals (Garcia-Macia et al., 2014; Guo et al., 2016; Cramer et al., 2018; Lee et al., 2022). Cramer et al. (2018) reported that callipyge lamb meat showed lower expression levels of cytochrome c and caspase 3 compared to normal lamb meat, which may lead to the delayed onset of apoptosis and consequently increased toughness. On the other hand, the occurrence of PSE pork was associated with higher expression levels of caspase enzymes due to their different apoptotic potentials compared to normal quality pork (Xing et al., 2018). However, there is limited information describing whether apoptosis-related factors impact on the meat quality characteristics of broilers, particularly the development of PSE condition. Therefore, this study was designed to investigate the differences in the levels of apoptotic factors, including cytochrome c and caspases, of the PM muscle, and their relationship to meat and sensory quality characteristics in the PSE broilers compared to the normal quality broilers, to understand the association between apoptotic potential and quality variation.

## MATERIALS AND METHODS

### Animals and Sample Procedure

A total of 135 PM muscles from Ross 308 broiler carcasses (male; 4 wk of age; average live weight 1,587 ± 293 g) were used in the present study. At 15 min postmortem, both the left and right PM muscles were excised during standard slaughtering process at a commercial slaughterhouse in a cold room. The muscle pH was immediately measured from the left side breast. At the same fillets, muscle samples of approximately 20 g were taken for real-time polymerase chain reaction (**RT-PCR**) analysis, were immediately frozen using liquid nitrogen, and stored at –80°C. The remaining left and entire right fillets were rapidly cooled with ice-cold water to minimize muscle damage and stored at 4°C until further analysis.

After 24 h postmortem, the meat quality traits, including pH_24 h_, meat color, drip loss, and cooking loss, were measured using the muscle samples from the left side. The quality classes were determined using the values of the pH_24 h_ and lightness (L*) as a measure of the meat color (Carvalho et al., 2014). The reddish, firm, and non-exudative (**RFN**) quality group (N = 81) had a pH_24 h_ value of ≥5.7 and L* value ranging from 48 to 53, and the PSE quality group (N = 54) had a pH_24 h_ value of under 5.7 and L* value of ≥53. The right PM muscle samples at 24 h postmortem were stored at –20°C for the sensory evaluation.

### Meat Quality Characteristics

The muscle pH values (pH_15__min_ and pH_24 h_) were determined on the surface on PM muscle using a pH instrument equipped with a penetration probe (Testo 206-pH2, Test Inc., Lenzkirch, Germany). Color assessment was performed using a Minolta chromameter (CR-400, Minolta Camera Co., Osaka, Japan) and the color was expressed in terms of CIE values for lightness (L*), redness (a*), and yellowness (b*) (Commission Internationale de l'Eclairage, 1978). For water holding capacity (**WHC**), percentage of drip loss and cooking loss were measured with reference to Honikel (1998). Drip loss was calculated as the difference in sample weight before and after storage at 4°C for 48 h. To determine cooking loss, muscle samples were weighed before and after cooking, which involved being sealed in a polyethylene bag and heated in a water bath (80°C) until the core temperature reached 71°C. Cooking loss was expressed as a percentage of the initial weight of the muscle sample.

### Sensory Quality Characteristics

A total of 135 right breast samples were randomly selected by coding with a 3-digit number and evaluated over 27 sessions, with 5 samples per each session. The panelists training and sensory evaluations were performed at the Kyungpook National University (**KNU**) according to the guidelines of the American Meat Science Association (2015), and human ethics approval was granted by the Bioethics Committee of KNU (protocol number 2019-0027). Frozen muscle samples were thawed at 4°C for 18 h and then cooked by pan-frying using an induction cooker (CIR-IH300RGL, Cuchen, Cheonan, Korea) until the internal temperature reached 71°C. Cooked samples were cut into 1.3 cm^3^ cubes and provided to trained panelists. The panelists evaluated the samples for 3 sensory quality attributes, including tenderness, juiciness, and flavor intensity, which were assessed on a 9-point scale.

### Quantitative RT-PCR

Total RNA was isolated from the left PM muscles at 15 min postmortem, following the manufacturer's instructions. The quantity of total RNA was assessed by electrophoresis and normalized accordingly. Complementary DNA (**cDNA**) was synthesized using 1 ng of total RNA. The synthesized cDNA was used for quantitative RT-PCR to measure the expressions of *CYCS* (cytochrome c; forward 5′-CCC AGT GCC ATA CGG TTG AA-3′ and reverse 5′-CTC ACC CCA AGT GAT ACC TTT GT-3′), *CASP9* (caspase 9; forward 5′-AGA TGA AAC TTG CCG ACG TT-3′ and reverse 5′-CTT CAG AAC GGG CGT AAT GT-3′), *CASP3* (caspase 3; forward 5′-TGGCCCTCTTGAACTGAAAG-3′ and reverse 5′-TCC ACT GTC TGC TTC AAT ACC-3’), *CASP7* (caspase 7; forward 5′-CAT TTA TGG CAC CGA TGG AC-3′ and reverse 5′-CCG GTC CAG AGT CAG TTT GT-3′), and *glyceraldehyde-3-phosphate dehydrogenase* (***GAPDH***; forward 5′-CGT CCT CTC TGG CAA AGT CC-3′ and reverse 5′-AAG ATA GTG ATG GCG TGC CC-3′). RT-PCR was carried out using SYBR green dye (A25741, Applied Biosystems, Foster City, CA) using an ABI 730 real-time PCR instrument (Applied Biosystems). The comparative 2^−ΔΔCt^ method for relative quantification was used to calculate the relative gene expression. The housekeeping gene was *GAPDH* which was used to normalize the RT-PCR calculation.

### Statistical Analysis

The meat quality traits and levels of apoptosis-related factors in the meat quality classes were analyzed using a general linear mixed model (**GLM**; SAS Institute, Cary, NC). To assess the sensory quality characteristics, a GLM was produced, using the pH_24 h_ and L* values as a fixed effects and the trained panelists as a random effect. The probability difference option was set at 5% and used for calculating the significant differences in the least-squares means (**LSM**) of investigated parameters between the groups. All data were presented as LSM with standard errors.

## RESULTS AND DISCUSSION

The breast meat and sensory quality characteristics of the meat quality classes are presented in Table 1. As expected, the RFN group exhibited higher muscle pH_15 min_ (6.38 vs. 6.27, *P* < 0.05) and pH_24 h_ (6.00 vs. 5.61, *P* < 0.001) values compared to the PSE group. A higher lightness value was observed in the PSE group than in the RFN group (56.8 vs. 49.6, *P* < 0.001), although redness and yellowness did not differ between the groups (*P* > 0.05). Breast muscle samples from the RFN group showed lower drip loss (2.19 vs. 2.66%, *P* < 0.05) compared to breast samples from the PSE group. Nam et al. (2009) reported that consumers are generally able to distinguish meat in unacceptable condition, especially PSE meat, due to its paler color and large amount of exudated water on the meat surface compared to meat in normal conditions (Livisay et al., 1996; Zequan et al., 2021). The occurrence of PSE meat is accompanied by economic losses for the meat industry as a result of its higher moisture loss and decreased purchases due to it's lower appearance acceptability (Kuttappan et al., 2012). However, when the cooked meat was evaluated by consumer panelists, the results differed among researchers. Livisay et al. (1996) reported a lower palatability score for the PSE compared to the RFN meat. However, Droval et al. (2012) and Nam et al. (2009) confirmed that consumers could not easily distinguish the differences in the organoleptic properties between the PSE and RFN meat. In the current study, the extent of water loss, especially cooking loss, was significantly different between the RFN and PSE groups (11.7 vs. 15.4%, *P* < 0.001). These differences in the WHC can significantly impact the palatability characteristics of cooked meat between the 2 groups, as cooking loss was negatively correlated with juiciness and tenderness scores. Trained panelists gave lower tenderness (5.29 vs. 5.88, *P* < 0.001) and juiciness (5.35 vs. 5.74, *P* < 0.001) scores for cooked samples from the PSE group compared to cooked samples from the RFN group, although there was no significant difference in flavor intensity between the two groups (*P* > 0.05). Therefore, the PSE chicken breasts tended to have impaired sensory quality compared to the RFN chicken breasts, a situation which may negatively affect the repurchase intentions of consumers for poultry meat.

**Table 1:** Comparison of meat quality and sensory quality characteristics of chicken breast between the meat quality classes.

	RFN (N = 81)	PSE (N = 54)	Level of significance
Meat quality characteristics			
pH_15 min_	6.38^a^ (0.03)^1^	6.27^b^ (0.04)	*
pH_24 h_	6.00^a^ (0.01)	5.61^b^ (0.02)	***
Lightness (L*)	49.6^b^ (0.25)	56.8^a^ (0.30)	***
Redness (a*)	1.41 (0.10)	1.22 (0.12)	NS
Yellowness (b*)	7.06 (0.19)	7.33 (0.23)	NS
Drip loss (%)	2.19^b^ (0.13)	2.66^a^ (0.17)	*
Cooking loss (%)	11.7^b^ (0.34)	15.4^a^ (0.39)	***
Sensory quality characteristics^2^			
Tenderness	5.88^a^ (0.09)	5.29^b^ (0.08)	***
Juiciness	5.74^a^ (0.08)	5.35^b^ (0.07)	***
Flavor intensity	6.29 (0.08)	6.25 (0.07)	NS

Abbreviations: RFN, reddish, firm, and non-exudative; PSE, pale, soft, and exudative.

Levels of significance: NS, no significant; * *P* < 0.05; *** *P* < 0.001.

a-b Different superscripts in the same row represent significant differences (*P* < 0.05).

1 Standard error of least-square means.

2 Score distribution (1‒9): tenderness, very tough to very tender; juiciness, not juicy to extremely juicy; flavor intensity, very weak to very strong.

**Figure 1:**
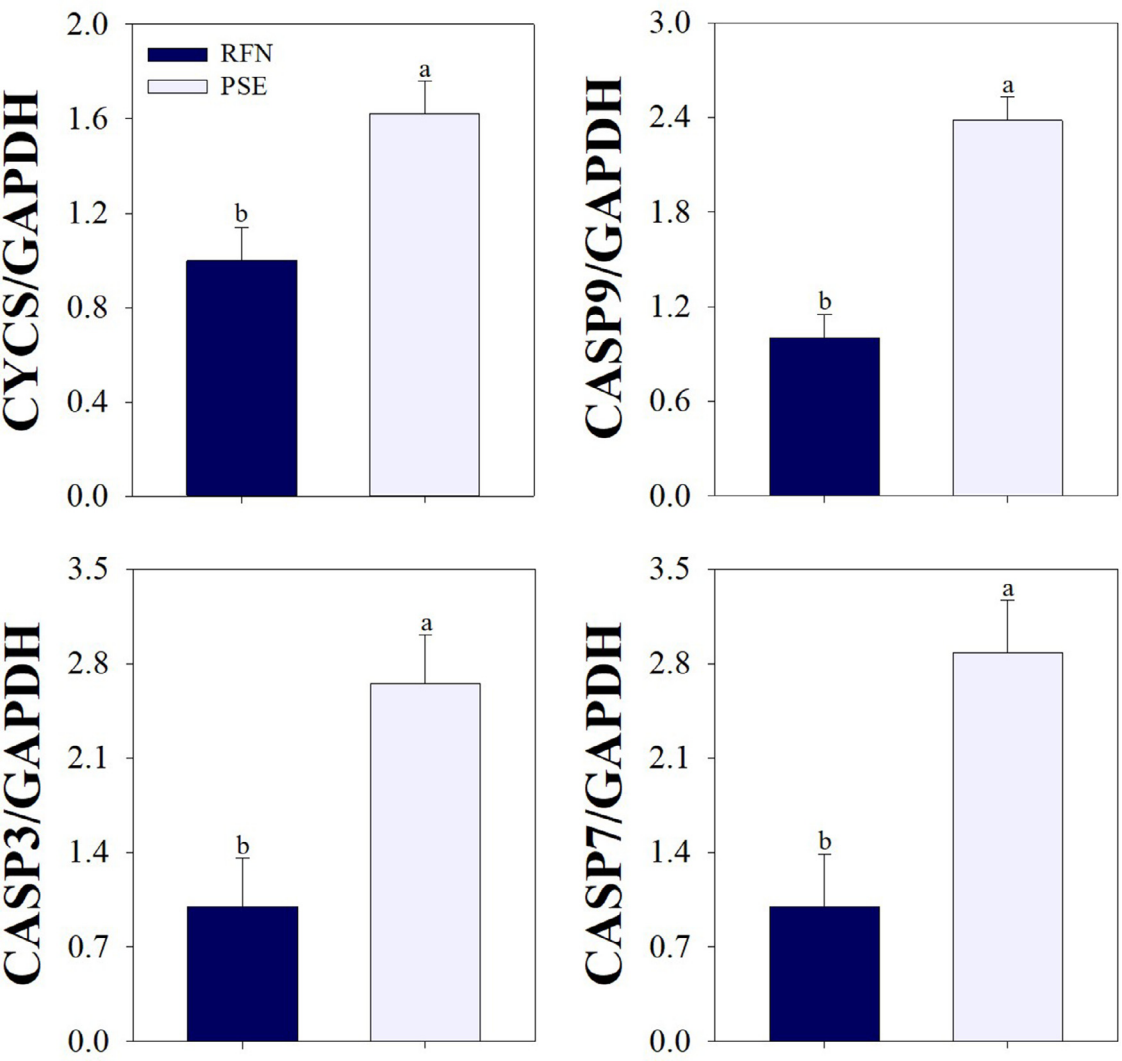
Quantitative RT-PCR for expression of cytochrome c (*CYCS*), caspase 9 (*CASP9*), caspase 3 (*CASP3*), and caspase 7 (*CASP7*) in the broiler *pectoralis major* muscle for the quality classes. *Glyceraldehyde-3-phosphate dehydrogenase* (*GAPDH*) was used to control for normalization. Bars indicate the standard error of the mean. ^a-b^Different letters denote significant differences (*P* < 0.05). Abbreviations: RFN, reddish, firm, and non-exudative; PSE, pale, soft, and exudative.

During conversion of muscle to meat after the exsanguination, the muscle metabolism changes. Aerobic metabolism is rapidly stopped and glycolysis becomes the primary form of metabolism as oxygen is depleted, and these conditions cause all muscle fibers to engage in cell death (Choi and Kim, 2009). Meanwhile, adenosine triphosphate (**ATP**) continues to be synthesized and utilized to sustain cellular homeostasis during postmortem glycolysis (Matarneh et al., 2021). Glycolytic metabolism during the postmortem period is causally linked to the caspase systems, as caspase proteases require ATP for activation in apoptotic muscle cells (Pradelli et al., 2014). Thus, alterations of ATP homeostasis in the postmortem muscle are affected by the extent of glycolysis and caspase activity, and associated with the accumulation of lactate and hydrogen ions, resulting in decreasing muscle pH at the early postmortem (Matarneh et al., 2021). Gorska and Wojtysiak (2018) reported that the caspase 3 activity at 30 min postmortem was significantly higher in the turkey breast muscles with lower muscle pH and higher lightness compared to muscles with higher muscle pH and lower lightness, as caspase 3 activity is accelerated by glycolytic metabolism (Secinaro et al., 2018; Ma et al., 2022). Thus, greater apoptotic and glycolytic potentials may increase the incidence of poultry meat under abnormal condition due to higher proteolytic activity and muscle acidification (Carvalho et al., 2014). However, there have been few studies on the relationship between the levels of other apoptosis-related molecules and chicken meat quality is limited. In the current study, the levels of various apoptosis-related factors were confirmed in chicken PM muscle at the early postmortem, and significant differences were observed between the quality classes (Figure 1). Cytochrome c, a key trigger of the apoptotic pathway, was highly expressed at 15 min postmortem in breast fillets showing PSE condition than in breast fillets showing RFN condition (1.62 vs. 1.00, *P* < 0.05); as a result, the RFN group had a lower level of initiator caspase 9 compared to the PSE group (1.00 vs. 2.38, *P* < 0.001). Additionally, muscle samples from the PSE group exhibited a higher level of caspase 3, which is the major caspase contributing to proteolysis in apoptotic cells, compared to those of the RFN group (2.65 vs. 1.00, *P* < 0.01). Caspase 7, which has a similar specificity profile as caspase 3, was present in lower levels in the RFN group than in the PSE group (1.00 vs. 2.88, *P* < 0.01). Thus, considering the levels of apoptotic factors in this study, the PSE condition chicken showed greater apoptotic potentials at the early postmortem period compared to the RFN condition chicken.

Taken together, these results indicate that the variation of meat quality in chicken PM muscles is associated with the rate of caspase-mediated apoptosis and glycolysis, which in turn are mediated by the levels of apoptosis-related factors at the early postmortem, and higher apoptotic potentials may lead to the PSE condition chicken breasts.
